# Label Retaining Cells (LRCs) with Myoepithelial Characteristic from the Proximal Acinar Region Define Stem Cells in the Sweat Gland

**DOI:** 10.1371/journal.pone.0074174

**Published:** 2013-09-18

**Authors:** Yvonne Leung, Eve Kandyba, Yi-Bu Chen, Seth Ruffins, Krzysztof Kobielak

**Affiliations:** 1 Eli and Edythe Broad CIRM Center for Regenerative Medicine and Stem Cell Research, University of Southern California, Los Angeles, California, United States of America; 2 Norris Medical Library, University of Southern California, Los Angeles, California, United States of America; 3 Department of Pathology, University of Southern California, Los Angeles, California, United States of America; National Cancer Institute, United States of America

## Abstract

Slow cycling is a common feature shared among several stem cells (SCs) identified in adult tissues including hair follicle and cornea. Recently, existence of unipotent SCs in basal and lumenal layers of sweat gland (SG) has been described and label retaining cells (LRCs) have also been localized in SGs; however, whether these LRCs possess SCs characteristic has not been investigated further. Here, we used a H2BGFP LRCs system for *in vivo* detection of infrequently dividing cells. This system allowed us to specifically localize and isolate SCs with label-retention and myoepithelial characteristics restricted to the SG proximal acinar region. Using an alternative genetic approach, we demonstrated that SG LRCs expressed keratin 15 (K15) in the acinar region and lineage tracing determined that K15 labeled cells contributed long term to the SG structure but not to epidermal homeostasis. Surprisingly, wound healing experiments did not activate proximal acinar SG cells to participate in epidermal healing. Instead, predominantly non-LRCs in the SG duct actively divided, whereas the majority of SG LRCs remained quiescent. However, when we further challenged the system under more favorable isolated wound healing conditions, we were able to trigger normally quiescent acinar LRCs to trans-differentiate into the epidermis and adopt its long term fate. In addition, dissociated SG cells were able to regenerate SGs and, surprisingly, hair follicles demonstrating their *in vivo* plasticity. By determining the gene expression profile of isolated SG LRCs and non-LRCs *in vivo*, we identified several Bone Morphogenetic Protein (BMP) pathway genes to be up-regulated and confirmed a functional requirement for BMP receptor 1A (BMPR1A)-mediated signaling in SG formation. Our data highlight the existence of SG stem cells (SGSCs) and their primary importance in SG homeostasis. It also emphasizes SGSCs as an alternative source of cells in wound healing and their plasticity for regenerating different skin appendages.

## Introduction

The skin contains a number of different skin appendages including hair follicles (hfs), sebaceous glands, nails, and sweat glands (SGs). In adult skin, each hf contains a reservoir of stem cells (SCs) localized in the bulge [1], [2], [3]. SCs reside in niches that provide a specialized environment to regulate their proliferation and differentiation which is important for tissue homeostasis and repair [4], [5], [6]. Not only are hair follicle stem cells (hfSCs) important for hf homeostasis and regeneration, they have also been shown to differentiate into epidermal cells during wound healing and so far, hfSCs are one of the best characterized systems in the skin [7], [8], [9].

In humans, however, eccrine SGs are present all over the body, more abundantly than hfs, with an important function to regulate body temperature. Improper thermoregulation can result in hyperthermia which could potentially lead to death. Eccrine SGs are coiled tubular glands that release their secretions through the straight duct at the distal part of SGs connected to the surface of the skin [10]. The secretory acinar part of SGs contains three distinct cell types: large basal clear cells rich in glycogen without secretory granules, small apical dark cells with Schiff-reactive granules, and myoepithelial cells localized between the basement membrane and basal part of clear cells. Clear cells are responsible for sweat secretion (water and electrolytes) through intercellular canaliculi which then reach the lumen through intercellular spaces between apical dark cells. Apical dark cells secrete glycoproteins into the lumen by exocytosis – a merocrine type of secretion [10], [11]. The normal sweat composition contains water, sodium, potassium, chloride, urea, creatine, creatinine, lactate and phosphate with small amounts of mucoprotein [10]. SG density varies in different regions of the body with its highest density (>200 glands/cm^2^) on the sole, palm and scalp in humans [12].

Furthermore, it has been shown that SG cells can contribute to and reconstitute a functional epidermis leading to great interest in this skin appendage as an alternative source of cells apart from hfSCs in skin regeneration [13], [14]. Previously, results suggested by Lobitz et al. in human skin showed that removal of the epidermis induced proliferation predominantly in basal cells of the SG straight ducts [15]. Subsequent experiments where SG ducts were injured showed an initial migration of lumenal cells after 24 h followed by proliferation of basal cells at 48 h giving rise to new spiraling lumenal cells as well as tongues of prickle cells at both ends of the cut duct [16]. Recently, Ritte et al. confirmed that eccrine SGs are the most abundant appendages in human skin and are major contributors of keratinocyte outgrowth to re-epithelialize the human epidermis after injury [17]. At the time we began this study, all previous reports have not addressed precisely which cell compartments or populations, if any, in SGs possess this regenerative capacity with stemness characteristic. Recently, however, Lu et al. have identified distinct stem cell populations in the basal and lumenal layers of sweat glands. Moreover, they and a previous group have shown that slow cycling, label retaining cells (LRCs) exist in the acinar part of sweat glands (SGs) possessing myoepithelial characteristic [18], [19]. However, to date, further molecular characterization of SG LRCs and whether these cells possess per se stem cell features have not been addressed.

Infrequently, slow dividing cells found in hair follicles and the corneal limbus have been previously described as stem cells [20], [21]. Therefore, this slow cycling characteristic has been suggested to be a common putative feature to identify SCs in different organs. In the skin, pulse chase experiments with labeled nucleotides designated the bulge as the residence of hf slow cycling, LRCs [7], [20], [22]. More recently, BrdU pulse chase experiments identified the presence of LRCs in the SG skin appendage [19], however, these methods were not useful to isolate live cells. A genetic approach which overcomes this obstacle was developed using the double transgenic mouse model with inducible expression of a histone 2B (H2B) conjugated to green fluorescent protein (GFP, H2BGFP) driven by a tissue specific keratin 5 (K5) transgene [23], [24]. In this double transgenic line, H2BGFP expression is turned on (“pulse”, no doxycycline treatment) from early embryogenesis. By feeding the animals doxycyline (Doxy), H2BGFP expression is turned off for the duration of the treatment (“chase”). During 4 weeks of “chase”, slow cycling cells retain H2BGFP expression (label retaining cells), whereas rapidly dividing transit amplifying cells dilute out the H2BGFP label upon each division. This approach was used to detect H2BGFP marked hf LRCs with slow cycling characteristic *in vivo* allowing the isolation and characterization of live hfSCs [24].

In this study, we exploited this H2BGFP LRCs system for the *in vivo* detection of infrequently dividing cells in SGs. This system allowed us to localize and isolate sweat gland stem cells (SGSCs) with label retaining characteristics. We observed that SG LRCs were restricted to the proximal acinar gland region and were not present in the SG ductal region. More specifically, LRCs were localized in the basal layer of the secretory acinar region and displayed myoepithelial characteristics consistent with the recent Lu et al. study [18]. While our data confirm the findings of Lu et al., our work here further strengthens these aspects of SG biology and in addition shows further molecular characterization of SG LRCs, which represent only a fraction of all SG basal layer cells recently isolated and characterized by Lu et al. [18]. Transcriptional analysis of SG LRCs and non-LRCs allowed us to define common and unique features of these populations *in vivo*. We also demonstrated that SG LRCs co-expressed keratin 15 (K15) in the acinar part and K15 labeled cells were able to survive long term and participate in SG homeostasis but not in epidermal homeostasis. Epidermal injury did not activate these SG cells in the proximal acinar region to participate in epidermal healing. However, when the system was further challenged under more favorable isolated wound healing conditions, we were able to activate quiescent SG LRCs to trans-differentiate into the epidermis. Furthermore, we demonstrated the plasticity of dissociated SG cells to regenerate SGs and, surprisingly, hair follicles *in vivo*. Understanding the biology of SGSCs may have great implications in developing potential treatments for patients with hyperhidrosis and patients with inherited anhidrotic or hypohydrotic ectodermal dysplasias who either lack or have underdeveloped appendages similar to burn victims [25], [26], [27], [28].

## Results

### Slow Cycling LRCs are Localized to the Proximal – Acinar Region of Sweat Glands

**Figure 1:**
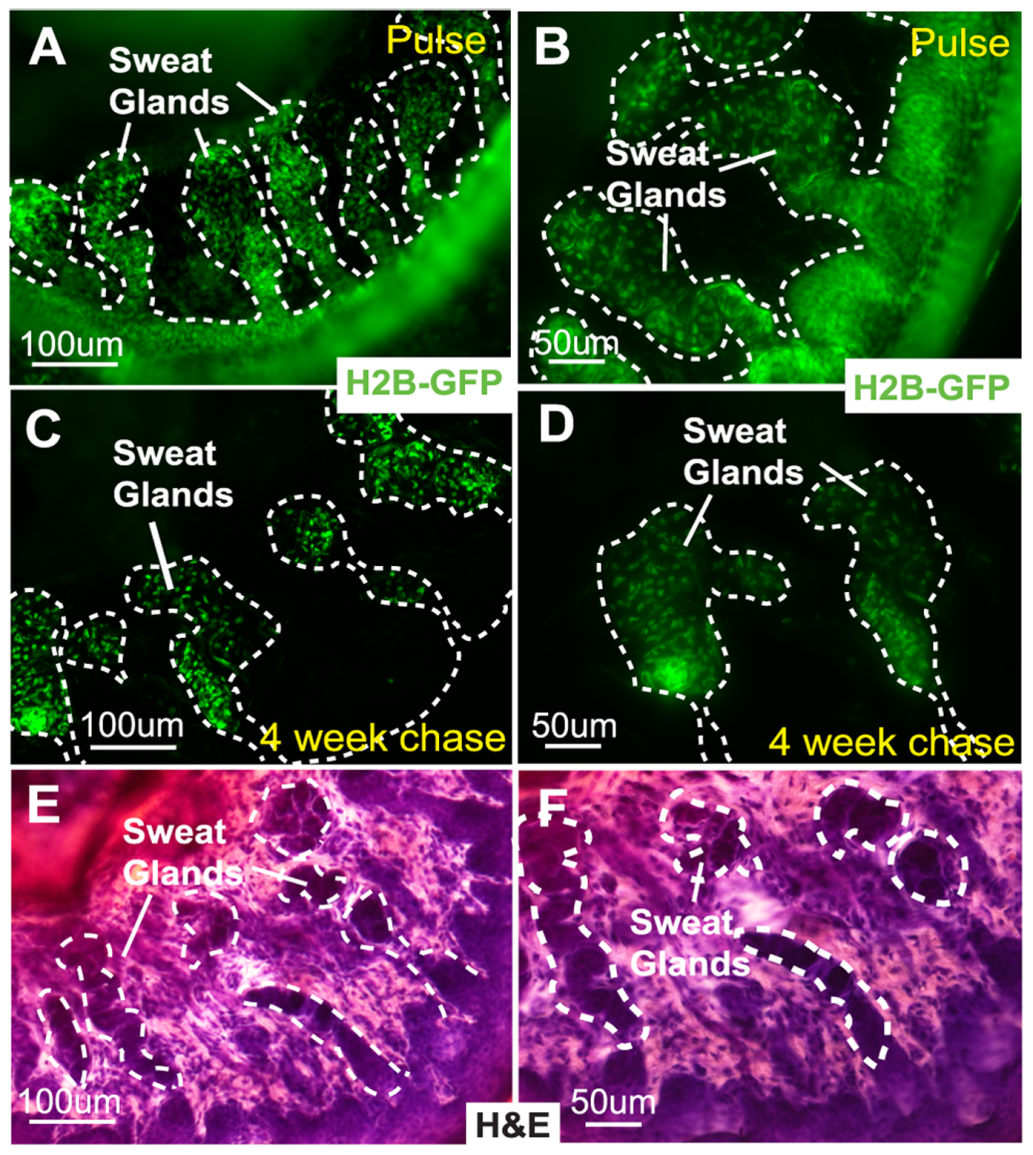
Sweat gland LRCs are localized in the acinar gland region of SGs. (A,B) H2BGFP is expressed in the epidermis and sweat glands before doxycycline treatment. (C,D) Sweat gland LRCs are found in the acinar gland region after 4 weeks of chase with doxycycline. (E,F) Tissue histology on sections with H&E staining. Abbreviations: LRCs, label-retaining cells; H2BGFP, histone 2B conjugated with green fluorescent protein; H&E, hematoxylin and eosin staining.

We employed the recently developed H2BGFP system, composed of two transgenic mouse lines: keratin 5-driven tetracycline repressor mice (K5-tTA) [23] and tetracycline response element-driven histone H2B-GFP transgenic mice (pTRE-H2B-GFP) [24], to detect live, slow cycling LRCs *in vivo*. In these animals, H2BGFP expression was uniformly detected in all cells of the epidermis, hf and SGs (ducts and glands) prior to Doxy treatment (Fig. 1A and 1B). After 4 weeks of “chase” experiments by switching off H2BGFP expression with Doxy treatment beginning at 3 to 4 weeks of age, we demonstrated the presence of infrequently dividing LRCs in SGs (Fig. 1C and 1D). These SG LRCs are extremely slow cycling with some of them persisting for more than 20 weeks of chase (Fig. S1). The H2BGFP expression of LRCs was restricted to the proximal acinar gland region with no expression in the ductal region (distal part of the SGs connected to the overlying epidermis) (Fig. 1C and 1D). This region was previously reported to contain LRCs in human eccrine glands [19]. As an internal control, we observed H2BGFP expression of LRCs restricted to hfSCs (bulge) after Doxy treatment (Fig. S1A). Histological localization of LRCs in SGs was confirmed on serial sections of the paw region by hematoxylin and eosin staining (H&E) (Fig. 1E and 1F).

### Sweat Gland LRCs are Attached to the Basement Membrane and Demonstrate Myoepithelial Characteristics

**Figure 2:**
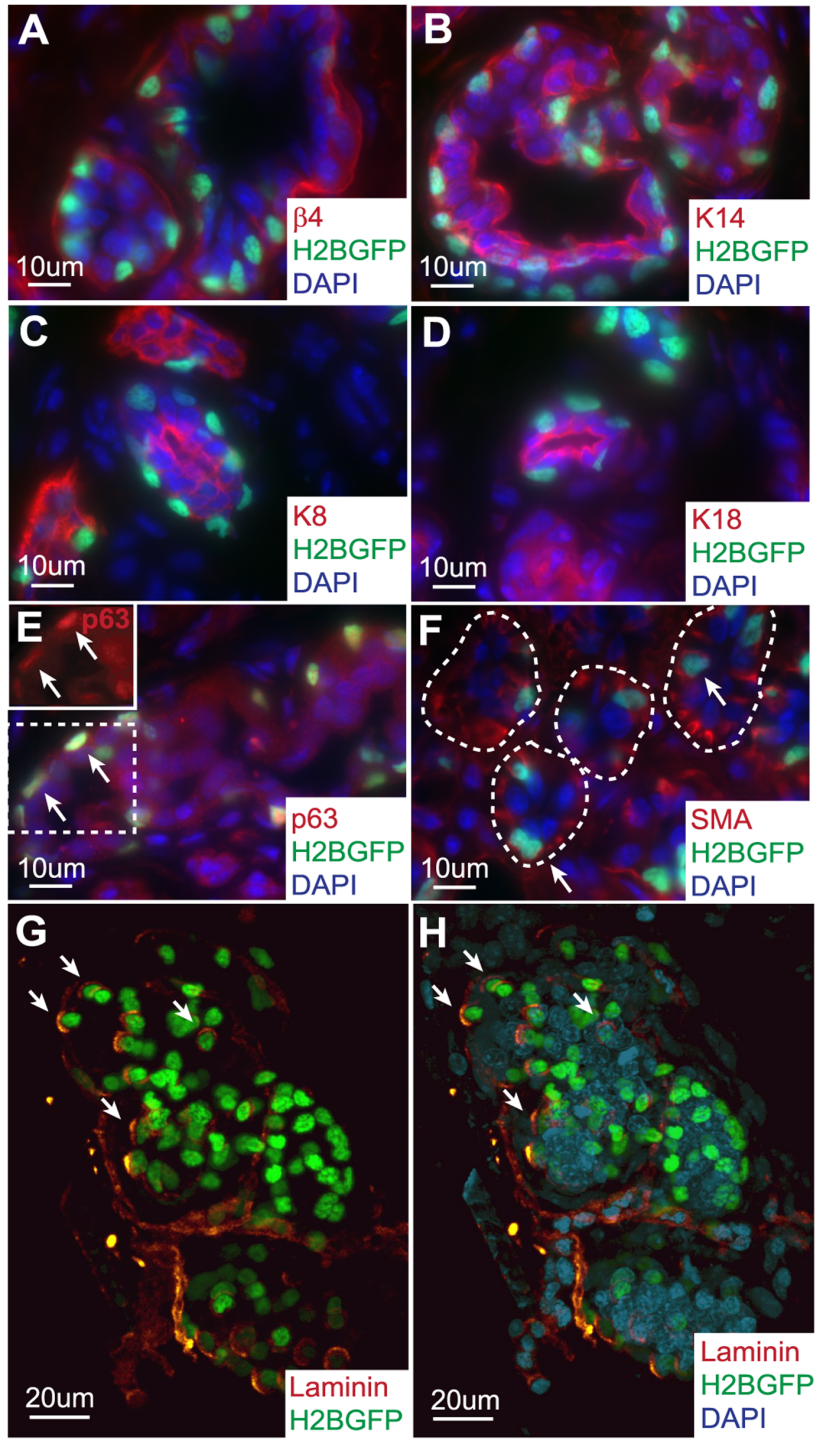
Sweat gland LRCs are attached to the basement membrane and possess myoepithelial characteristics. (A) Sweat gland LRCs are attached to the basement membrane positive for β4 integrin (red) and are (B) found in the basal layer co-localizing with K14. (C,D) Sweat gland LRCs do not express lumenal layer markers K8 and K18, respectively. (E) Within the basal layer, LRCs co-localize with myoepithelial cell marker p63, inset denotes p63 single channel, arrows and (F) with myoepithelial cell marker smooth muscle actin (SMA), arrows. (G,H) Whole mount staining of SGs with laminin with and without DAPI. Arrows denote co-localization of markers with H2BGFP marked LRCs. Abbreviations: LRCs, label-retaining cells; H2BGFP, histone 2B conjugated with green fluorescent protein; SMA, smooth muscle actin; K, keratin.

SGs are composed of three different cell types, dark apical cells of the lumen, clear and myoepithelial cells of the basal layer. Therefore, we used immunofluorescence staining with a number of different markers to determine where SG LRCs are localized. We demonstrated that these SG LRCs are attached to the basement membrane expressing β4 integrin (Fig. 2A). In addition, these SG LRCs also co-expressed the basal layer marker keratin 14 (K14) (Fig. 2B), whereas lumenal layer markers, keratin 8 (K8) and keratin 18 (K18), did not overlap with SG LRCs (Fig. 2C and 2D, respectively). Next, by performing p63 antibody (Ab) staining for a mammary gland myoepithelial cell marker [29], which has also been shown to be important for epidermal self-renewal and differentiation [30], we distinguished that SG LRCs specifically co-localized with myoepithelial cells of the basal layer (Fig. 2E). In addition, we show co-localization of smooth muscle actin (SMA) with SG LRCs, which further support that SG LRCs have a myoepithelial cell characteristic with basal layer localization (Fig. 2F).

SGs exist as 3-dimensional (3D) structures, therefore, we performed whole mount staining of 4 weeks chased SGs with a basement membrane marker, laminin, (with DAPI nuclear counterstain) to examine how these LRCs are organized within this appendage (Fig. 2G and 2H). We used two-photon confocal microscopy to acquire serial Z-stacks and performed 3D re-construction of enzymatically purified whole SGs (see Material and Method for details). This allowed us to generate a 3D model of SGs to visualize the 3D organization of LRCs attached to the basement membrane organized in a scattered fashion (Fig. 2G and 2H, arrows, Movie S1). This contrasts with the clustered LRCs organization found in the hair follicle and cornea limbus.

### Sweat Gland LRCs Express Keratin 15 (K15) in the Acinar Region and K15 Marked Cells Contribute Long Term to the Sweat Gland Structure but not to Epidermal Homeostasis

**Figure 3:**
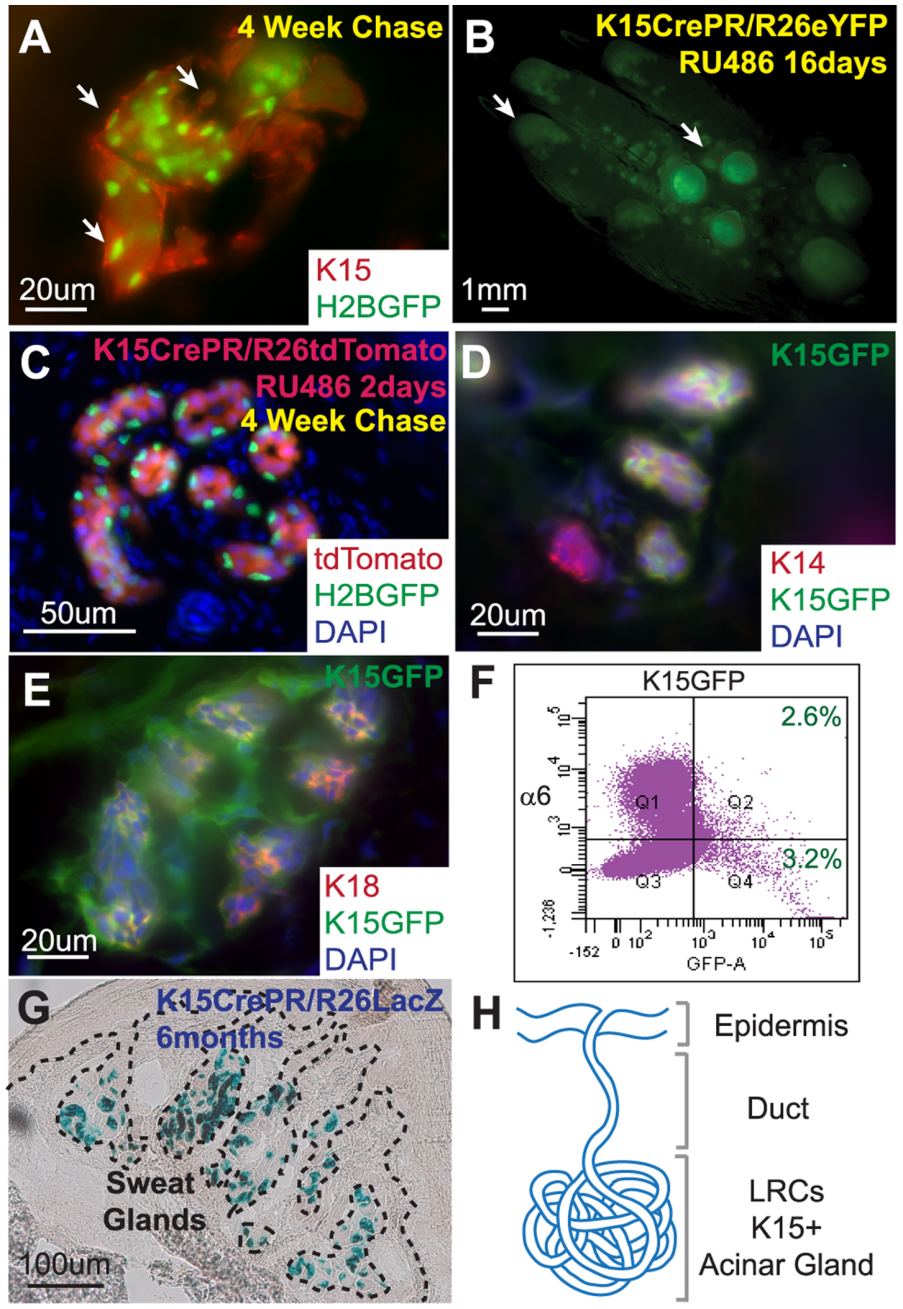
Sweat gland LRCs express Keratin 15 and contribute long term to the acinar SG structure. (A) K15 staining of sweat gland LRCs indicate positive K15 expression. (B) Fluorescent photo of K15CrePR/R26eYFP^RU^ palm containing YFP positive sweat glands after long term YFP activation. (C) Section of K15CrePR/R26tdTom^RU^ crossed onto K5TetOff/TreH2BGFP sweat glands after 4 weeks of chase with doxycycline followed by 2 days of RU treatment. (D) K14 basal layer staining co-localizes with GFP expression in K15-GFP transgenic sweat glands. (E) K18 lumenal marker staining co-localizes with K15-GFP expression in sweat glands. (F) FACS analysis of K15-GFP sweat glands demonstrates that approximately half of the K15-GFP positive cells are localized to the basal layer expressing α6 integrin. (G) Histology of X-Gal-treated K15CrePR/R26LacZ transgenic mice, blue stain indicates transgene expression for more than 6 months after RU activation in sweat glands. (H) K15 expression co-localizes with LRCs in the acinar sweat gland region.

It has been previously shown that another LRC population located in the hf bulge specifically expresses K15 [3], therefore, we examined whether K15 expression is also present in SGs. First, using K15 Ab staining, we demonstrated that mouse SG LRCs co-localized with K15 (Fig. 3A) confirming previously published data for human eccrine gland LRCs [19]. Next, we established a genetic lineage tracing approach to monitor K15 expressing cells in SGs by generating a K15 driven Cre recombinase conjugated with a truncated progesterone receptor (PR), (K15CrePR) [3], with three different Cre-dependent Rosa26 reporter mice: eYFP (enhanced Yellow Fluorescent Protein, R26eYFP) [31], tdTomato (tandem dimer Tomato, R26tdTom) [32] and LacZ (β-galactosidase, R26LacZ) [33]. After RU486 (RU) treatment for 16 days beginning at P43, YFP expression was detected in SGs of the palm in both the foot pads and fingertips (Fig. 3B, arrows). This system allowed us to mark initially K15 positive cells and permanently label all of its progeny in SG structures.

To demonstrate whether K15 marked cells in SGs co-localized with LRCs, we used a K15CrePR system with a Rosa26-tdTomato reporter mouse crossed onto the K5TetOff/TreH2BGFP background. After 4 weeks of chase with Doxy treatment (at ∼P49) when LRCs (green) were present in SGs, we labeled the K15 expressing cells using a short RU treatment for 2 days to mark K15 positive cells in SGs with tdTomato expression (Fig. 3C). These results revealed that tdTomato expression (red) was restricted to the acinar regions of the SGs and co-localized with LRCs in the basal layer (Fig. 3C). We also used a K15-GFP (Green Fluorescent Protein) reporter system [3] to visualize cells which actively expressed K15 (without permanently labeling their progenies) and confirmed that K15-GFP expressing cells were localized in the acinar region of SGs (Fig. 3D and 3E) where LRCs are found (Fig. 3A and 3C). Although K15 expression includes the LRCs population, they are not specific to SG LRCs. We stained K15-GFP SGs for the basal layer marker, K14, and the lumenal layer marker, K18, to demonstrate that K15 positive SG cells marked both basal and lumenal layers of the SG structure in adult mice (Fig. 3D and 3E). Additionally, fluorescence activated cell sorting (FACS) analysis from K15-GFP mice demonstrated that less than 6% of the total number of isolated cells from SG regions were GFP positive (Fig. 3F). Approximately less than half of the K15-GFP positive cells (2.6% out of 5.8%) were labeled by α6-integrin, a basal layer marker, with the remaining half (3.2% out of 5.8%) of K15-GFP positive SG cells negative for α6-integrin (Fig. 3F), which are presumably K15 positive lumenal cells. Previously, Lu et al. have used the same K15CrePR promoter to identify progenitor cells of the lumenal layer [18]. However, K15 expression was activated at an early postnatal stage (P5) prior the completion of SG morphogenesis. Here, we analyzed K15 expression in mature SGs of adult mice and showed that K15 targets the lumenal layer of SGs consistent with the previous report. But, by inducing K15 expression in adult mice, we show that K15 can also target basal layer cells in addition to lumenal cells. Finally, to address the long term contribution of K15 expressing cells in SG and epidermal homeostasis, we used K15CrePR mice crossed with a R26LacZ reporter. After induction of LacZ by 16 days of RU treatment in adult mice, we were able to detect LacZ positive cells (with X-gal staining) from the time of RU treatment to more than 6 months after initial transgene activation in the acinar region (Fig. 3G). This demonstrated that K15 positive SG cells could survive long term and contribute to SG homeostasis. However, LacZ positive cells were not present in the surrounding epidermis or distal SG duct (Fig. 3G) suggesting that these cells do not contribute towards physiological epidermal homeostasis (Fig. 3H) behaving similar to hfs [8].

### Isolating LRCs from Sweat Glands

**Figure 4:**
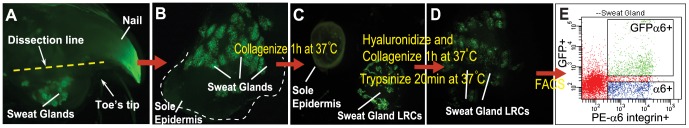
Isolation strategy for sweat gland LRCs. (A) Whole K5TetOff/TreH2BGFP toe tip under the stereomicroscope, dotted yellow line marks sweat gland dissection. (B) Dissected sweat glands with surrounding sole’s epidermis. (C) Separation of sweat glands from attached sole’s epidermis after collagenase treatment. (D) A series of further enzymatic digestions result in a single cells suspension of sweat gland cells for FACS. (E) Sweat gland cells sorted for H2BGFP and α6 integrin-PE double positive cells as well as α6 integrin-PE single positive surrounding basal cells.

To isolate pure fractions of SG LRCs, we used a combination of surgical dissection with subsequent enzymatic digestions. To avoid contamination from hf LRCs, we collected the whole paw with the toes and dissected out SGs with the surrounding sole’s epidermis (Fig. 4A, yellow dissection line and Fig. 4B). To purify SGs from the attached sole’s epidermis, we then digested the dissected SGs with collagenase for 1 hour at 37°C and mechanically separated the epidermis (Fig. 4C). Purified SGs were further digested with collagenase and hyaluronidase at 37°C for 1 hour and then trypsinized for 20 min at 37°C to obtain a single cell suspension ready for FACS (Fig. 4D). Since SG LRCs were attached to the basement membrane, we stained these cells with a FACS specific antibody against α6 integrin to help purify these live LRCs and adjacent non-LRC basal cells. The specific FACS gates to sort double positive SG LRCs (H2BGFP+ and α6 integrin+) and single positive SG non-LRCs (H2BGFP- and α6 integrin+) were setup according to negative (unstained), single GFP positive and single α6 integrin positive control cells (Fig. 4E). The majority of SG LRCs (H2BGFP+) were positive for α6 integrin (Fig. 4E). In parallel, we isolated adjacent SG non-LRC basal cells (α6 integrin+) (Fig. 4E). FACS analysis revealed that SG LRCs accounted for approximately 1–6% of the whole dermal fraction containing the SG skin appendage (after detachment of the sole’s epidermis).

### Defining the Sweat Gland LRCs Characteristic

**Figure 5:**
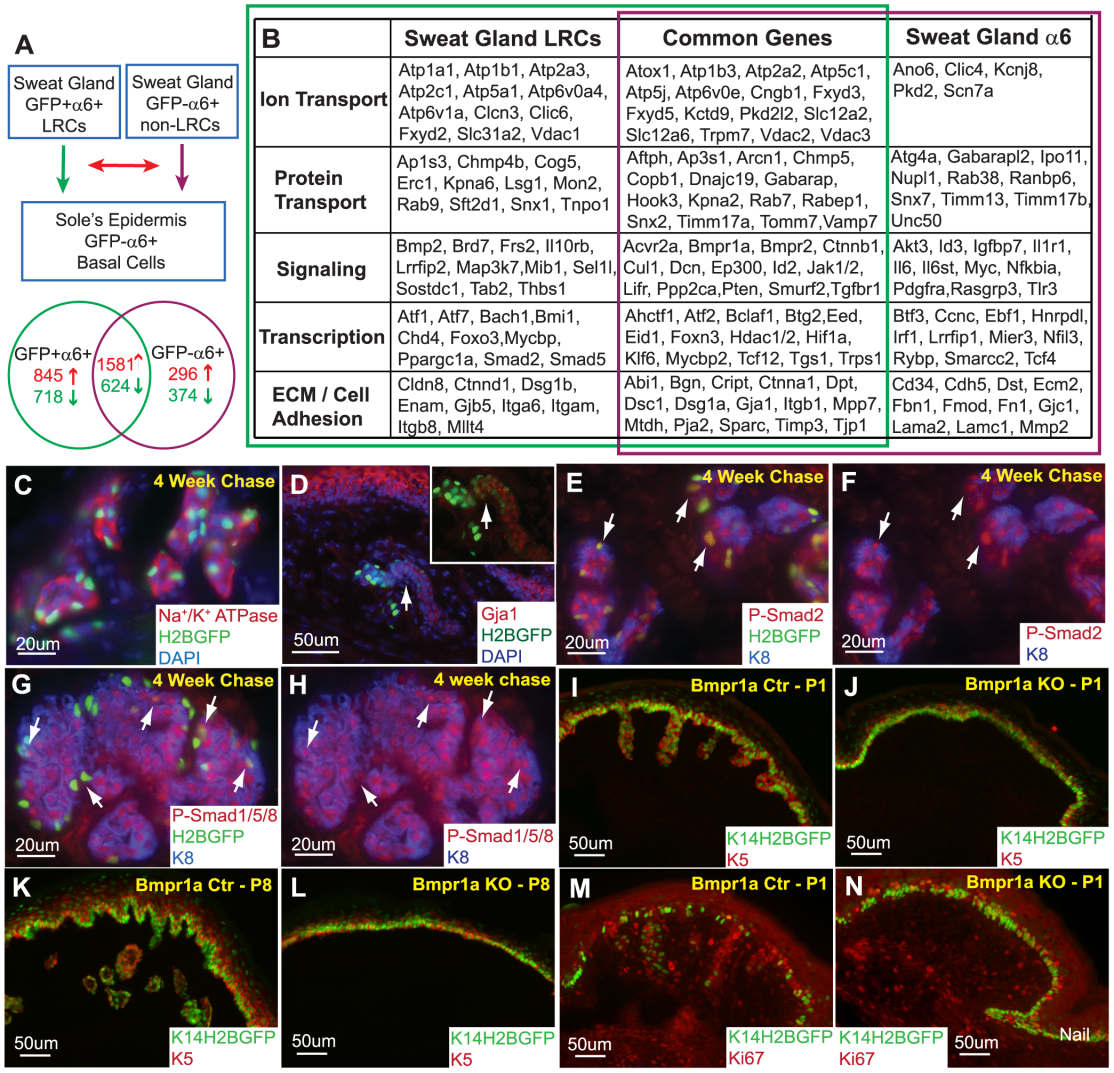
Molecular characteristics of sweat gland LRCs define BMP signaling as a requirement for SG formation. (A) GFP+/α6+ sweat gland LRCs and GFP−/α6+ sweat gland non-LRCs basal cells were compared to the basal layer of the sole’s epidermis. The resulting gene expression profiles of these sweat gland LRCs and basal cells were then compared to each other. (B) Gene expression profiles consistently found in two independent microarray analyses from independent biological samples were categorized into ion and protein transport, signaling, transcription, extracellular matrix (ECM) and cell adhesion based on function. (C) Sodium Potassium ATPases were confirmed to be expressed in the sweat glands. (D) Gja1 is confirmed to be expressed in SG LRCs (arrows) as well as non-LRCs. (E) Phospho-Smad2 co-localizes with SG LRCs, arrows. (F) Corresponding phospho-Smad2 and K8 channels. Arrows indicate LRCs marked in panel “E”. (G) Positive phospho-smad1/5/8 staining indicates active BMP signaling in sweat glands. (H) Corresponding phospho-Smad1/5/8 and K8 channels with arrows indicating co-localization with some LRCs. (I) Downgrowth of sweat glands is observed in P1 control paws but is absent (J) in Bmpr1a/K14Cre/K14-H2BGFP KO paws. (K) Similarly, more developed sweat glands are observed in P8 control paws but are still absent (L) in KO mice. (M) The basal layer of the epidermis as well as the sweat glands is proliferative at P1. (N) Although sweat glands are absent in Bmpr1a/K14Cre/K14-H2BGFP KO paws, the epidermal basal layer is still capable of division.

To identify the transcriptional gene expression profile of SG basal layer cells, total RNA was extracted from the GFP+/α6+ population (SG LRCs), GFP−/α6+ adjacent basal layer cells (SG non-LRCs), and α6+ basal layer cells of the sole’s epidermis for microarray analyses from two independent experiments. Purification and microarray hybridization (Affymetrix Mouse Gene 1.0ST) of each fraction were performed in duplicate. Then, each population, SG LRCs and non-LRCs, were compared separately to the basal layer of the sole’s epidermis (Fig. 5A). By comparing SG LRCs to the sole’s epidermal basal layer (α6+), we identified 2426 (845+1581) genes to be consistently up-regulated and 1342 (718+624) genes to be down-regulated by at least 2-fold in two independent microarray analyses. Subsequently, we compared non-LRCs to the sole’s epidermal basal layer (α6+) and identified 1877 (296+1581) genes to be consistently up-regulated and 998 (374+624) genes down-regulated by at least 2-fold (Fig. 5A). We then examined how many of the gene changes identified in SG LRCs and SG non-LRCs were commonly or uniquely expressed between both populations. We identified 1581 up- and 624 down-regulated genes (a total of 2,205 genes) to be commonly up-regulated or down-regulated by at least 2-fold in two independent microarray analyses from both GFP+/α6+ and GFP−/α6+ basal layer cells of the SG (Fig. 5A and Table S1). Moreover, 1563 genes were uniquely either up- (845) or down- (718) regulated in SG LRCs (Fig. 5A and Table S2), whereas 670 genes were uniquely up- (296) and down- (374) regulated in SG non-LRCs (Fig. 5A and Table S3). All these genes were consistently changed in the two independent experiments conducted. Some of the genes were validated through real-time PCR using separately isolated biological samples from 2–3 independent experiments. In particular, genes expressed in the SG GFP-α6+ non-LRCs, Mmp2 and Timp2, were confirmed to be up-regulated through real-time PCR when compared to the SG LRCs fraction (Fig. S2). In addition, we also performed real-time PCR for biglycan which was found to be up-regulated in both SG LRCs and non-LRCs in the microarray analyses. Although biglycan was found to be commonly expressed in both fractions, it was up-regulated by at least 2-fold more in the non-LRCs fraction compared to the LRCs population. Accordingly, our real-time PCR data validates that biglycan is up-regulated in SG GFP-α6+ non-LRCs when compared to SG LRCs. All real-time data was performed in triplicate using cDNA from either 2 or 3 independent biological samples.

As SG LRCs showed myoepithelial characteristics, we further probed how the gene expression profile in this population corresponded to its function when compared to SG non-LRCs basal cells. To this end, we performed functional annotations (grouped according to the DAVID software) which enabled us to categorize a number of identified genes in LRCs and non-LRCs (Fig. 5B). We found that a number of these genes were involved in cell adhesion, signaling and transcription. Moreover, a number of transporters and ion channels, important for SG function, were identified in the microarray analyses (Fig. 5B). In particular, we identified the expression of sodium/potassium (Na^+^/K^+^) ATPase pumps (Atp1a1, Atp1b1, Atp1b3) in both GFP+/α6+ and GFP−/α6+ cells of the SGs. This expression was confirmed by Na^+^/K^+^ ATPase Ab staining in the SG skin appendage after a 4 weeks chase with Doxy (Fig. 5C). In addition, vimentin was found to be commonly up-regulated in LRCs and non-LRCs, which was previously reported in the human SG myoepithelium [34]. To further validate the genes identified in the microarray, we stained 4 weeks chased SGs with gap junction protein, alpha 1 (Gja1), which was up-regulated in both SG LRCs and non-LRCs, and confirmed its co-localization with SG LRCs (Fig. 5D, arrow) and non-LRCs of the sweat duct. Positive phospho-Smad2 (p-Smad2) staining in SG LRCs and lumenal cells (indicated with K8 co-expression) also confirms its up-regulation in the microarray (Fig. 5E and 5F). Furthermore, a number of genes important for Bone Morphogenetic Protein (BMP) signaling, including Bmpr1a, Bmpr2, Smad5, Id2, Id3 and Decorin were up-regulated in the SG when compared to the sole’s epidermis (Fig. 5B). Therefore, we tested for BMP signaling activity using phospho-smad1/5/8 staining and observed positive nuclear phospho-Smad activity in adult mouse SGs suggesting that BMP signaling may be important in this appendage. More specifically, we show nuclear phospho-smad1/5/8 expression in some LRCs (Fig. 5G and 5H, arrows). However, its expression was not specific to LRCs and was also found in other SG cells including lumenal cells. To functionally examine the effects of BMP signaling, we ablated Bmpr1a during development using a keratin 14 driven Cre recombinase (K14Cre) [35] crossed onto a K14-H2BGFP reporter [36]. At P1 and P8, we observed downgrowth and SG development in control mice (Fig. 5I and 5K). However, in Bmpr1a knockout (KO) mice, SGs were absent and failed to form suggesting that BMP signaling is required for normal SG morphogenesis (Fig. 5J and 5L). Ki67 staining indicated that the basal layer of Bmpr1a KO epidermis appeared capable of proliferation, but failed to acquire the SG fate (Fig. 5N vs. control 5M).

### Acinar Sweat Gland Cells Do Not Contribute to the Epidermis during Wound Healing

Previously, it has been reported that bulge hfSCs with LRCs characteristic do not participate in epidermal homeostasis, but they can actively deliver cells to the epidermal wound during skin injury [8]. In addition, keratinocytes in the region directly above bulge LRCs, marked by Lgr6, can postnatally generate the sebaceous gland and interfollicular epidermis contributing to epidermal homeostasis and can execute long term wound repair [37], [38]. However, little is known about the role of SGs in active epidermal regeneration initiated upon wounding. In human SGs, it has been reported that basal cells of the straight duct undergo division when provoked by skin injury [15].

**Figure 6:**
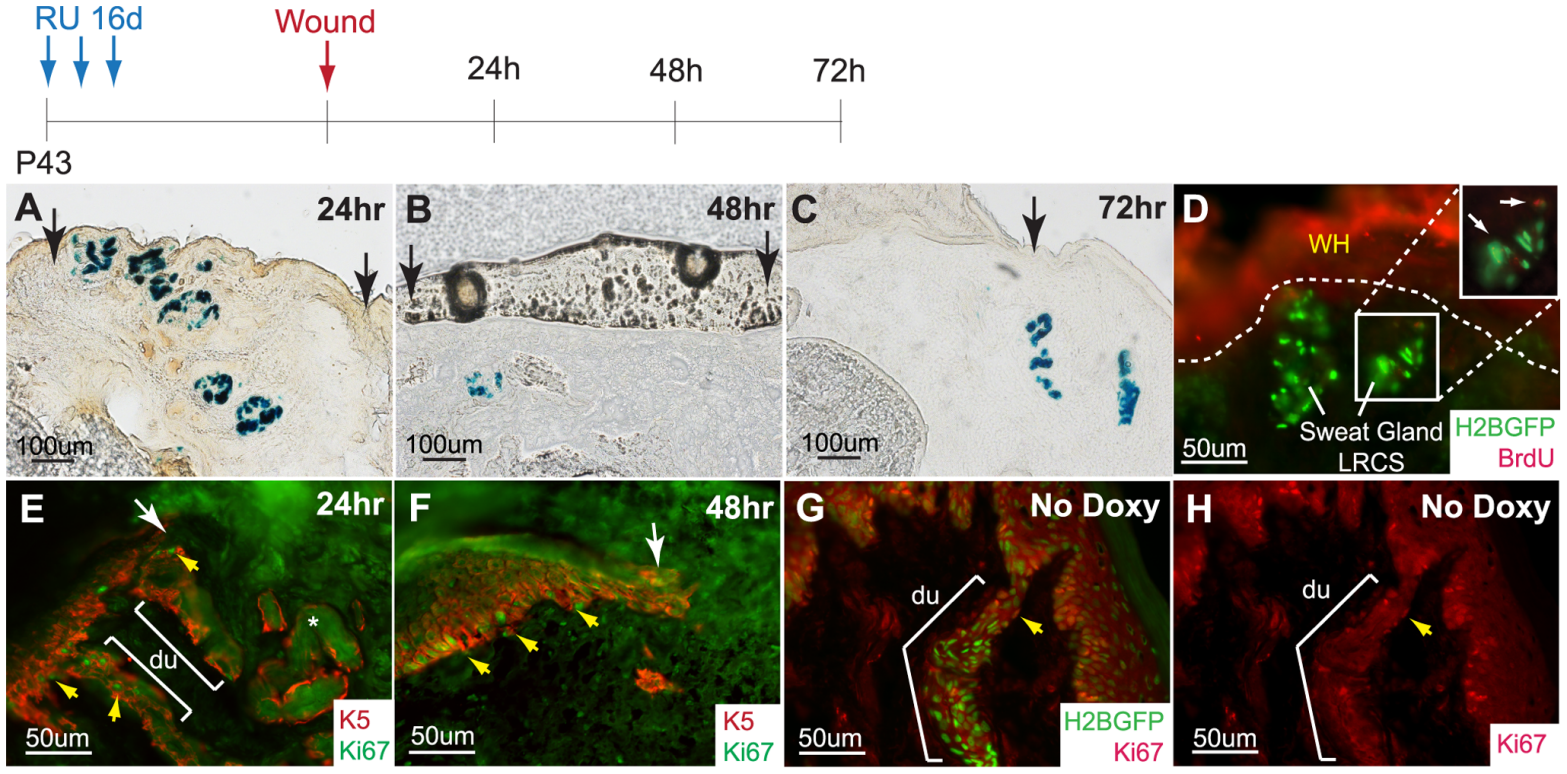
Acinar sweat gland cells do not contribute to the epidermis during typical wound healing. (A) K15CrePR/R26LacZ^RU^ marked sweat gland cells do not contribute to the epidermis at 24 h, (B) 48 h, and (C) 72 h after wounding. (D) BrdU pulse shows that a few SG cells are activated upon injury (inset, arrows) while most SG LRCs remain quiescent. (E) Ki67 staining confirms that the acinar sweat gland region is quiescent while the SG duct and epidermal basal layer is proliferative at 24 h and (F) 48 h. (G) Under normal homeostasis, cells of the SG duct and epidermal basal layer are active in the cell cycle. (H) Corresponding single Ki67 (red) channel. Abbreviations: du, sweat ducts.

Since we have demonstrated that SG acinar cells marked by K15CrePR/R26LacZ do not participate in epidermal keratinocyte lineages during homeostasis, we next examined if these acinar SG cells could respond and actively contribute to epidermal wound repair upon injury. Wounds, where the epidermis was effectively scraped off, were performed on K15CrePR/R26LacZ mice in order to trace K15 positive SG cells and their progenies. Wounds were allowed to heal for 24 h, 48 h, and 72 h when samples were collected for analysis. X-gal staining for LacZ enabled visualization of K15 positive SG cells and their progeny (blue). At all time points, no blue cells were detected in the regenerating epidermis (Fig. 6A-C). To investigate whether SG LRCs were responding to the injury, we performed similar wound healing experiments on 4 weeks chased K5TetOff/TreH2BGFP mice. In this experiment, we pulsed the animals with BrdU to probe for proliferating cells in SGs. BrdU was detected sporadically in a few LRCs, but most SG cells remained quiescent (Fig. 6D). To examine which cells were responding to the injury, we stained the wound healing samples from K15CrePR/R26LacZ mice with a Ki67 Ab and found that the basal layer of the epidermis and SG ductal cells were active in the cell cycle, whereas the SG acinar region remained quiescent (Fig. 6E and 6F). Under physiological homeostasis, SG duct cells appear to be more active than the SG acinar region similar to the basal layer of the epidermis (Fig. 6G and 6H).

### Sweat Gland LRCs can Trans-Differentiate into the Epidermis under Prolonged Isolated Wound Healing

**Figure 7:**
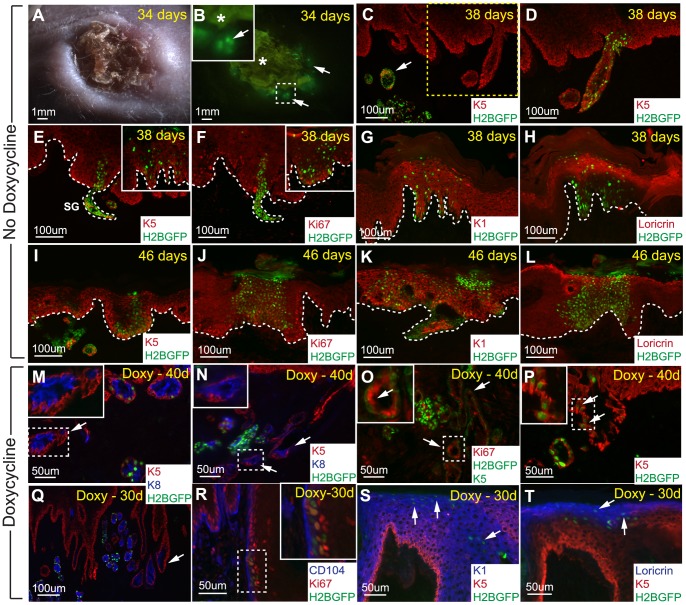
Sweat gland LRCs can trans-differentiate into the epidermis under prolonged isolated wound healing conditions. (A) DIC photo of the back skin area containing transplanted H2BGFP labeled sweat glands at 34 days. (B) Corresponding fluorescent image displaying the presence of H2BGFP positive cells in the grafted area. Arrows indicate regions with H2BGFP positive cells while “*” marks autofluorescence of the wounded area. (C) Biopsy was taken at 38 days where H2BGFP sweat glands were observed in the dermis, arrow. (D) Higher exposure time for the GFP channel on the same section from “C” detected marked cells with lower H2BGFP intensities in the epidermis. (E,I) K5 basal layer staining demonstrating the contribution of sweat gland cells to the newly formed epidermis at 38 and 46 days, respectively. (F,J) These H2BGFP labeled cells are still able to proliferate as indicated by Ki67 staining. (G,K) Sweat gland cells can differentiate into cells of the suprabasal layer marked by K1 at 38 and 46 days, respectively. (H,L) These sweat gland cells can also contribute to the granular layer as marked by loricrin. (M,N) When 4 weeks chased sweat glands are transplanted and kept on doxycycline treatment, the H2BGFP label gets diluted out of some sweat glands (arrows), as confirmed by K5 and K8 staining for sweat glands. (O) Ki67 staining show that these sweat glands lacking visible H2BGFP LRCs (green - nuclear) defined by K5 positive staining (green membrane staining) contains Ki67 positive dividing cells (arrows). (P) H2BGFP sweat gland LRCs can contribute to the newly formed epidermis as indicated by K5 basal layer staining (arrows). (Q) Some sweat glands are connected to the newly formed epidermis lacking visible H2BGFP LRCs. (R) Sweat gland LRCs contributing to the epidermis are proliferative near the CD104 marked epidermal basal layer as marked by Ki67, inset shows magnification of co-localization. (S) Sweat gland LRCs can contribute to the suprabasal layer marked by K1 as well as the (T) granular layer expressing loricrin. White dotted lines mark dermal-epidermal interfaces. Abbreviations: CD104, β4 integrin.

Although the SG acinar cells did not contribute to wound healing under normal circumstances, we further challenged the system using more favorable conditions. For this, we isolated 4 weeks chased H2BGFP labeled whole SGs through collagenase digestion (as described in Fig. 4B–C and Material and Method). Then, we transplanted the dermal portion of these SGs (without sole’s epidermis) into a silicon chamber implanted onto the backs of immunocompromised “nude” mice [39]. The advantage of this chamber graft experiment was that the transplanted dome containing the SGs physically separated and initially prevented the surrounding “nude” epidermis from closing the wound, thereby increasing the chance for the transplanted SG epithelial cells to respond to wound regeneration. The chamber was removed approximately 14 days after transplantation and the wound was allowed to heal for about one month (Fig. 7A). At 34 days after transplantation, H2BGFP labeled cells were observed in the transplanted region of the regenerated skin (Fig. 7B). To determine the participation of these marked SG cells in epidermal regeneration, we sectioned and stained these transplants with a number of different markers at 38 and 46 days after transplantation. The majority of the transplanted H2BGFP marked SGs were found in the dermis (Fig. 7C, arrows). However, when we closely examined the epidermis and increased exposure time for the GFP channel on the same section, we found that a number of H2BGFP expressing cells with lower intensity contributed to the newly formed epidermis (Fig. 7C vs. 7D). These H2BGFP cells co-localized with the basal layer marker, keratin 5 (K5), at 38 and 46 days (Fig. 7D, 7E and 7I, respectively). Furthermore, we demonstrated that these H2BGFP labeled SG cells could proliferate along the basal layer using Ki67 co-staining (Fig. 7F and 7J). Next, we checked if the H2BGFP marked SG cells found in the regenerating epidermis were able to adopt epithelial characteristic by performing immunofluorescence staining for different epidermal differentiation markers. At both 38 days and 46 days, we showed that the H2BGFP positive SG cells can contribute to the suprabasal layer of the epidermis indicated by co-localization with K1, a suprabasal layer marker (Fig. 7G and 7K). Similarly, we observed that H2BGFP labeled cells could differentiate into cells of the granular layer indicated by loricrin staining (Fig. 7H and 7L). Thus, these lineage tracing experiments demonstrated that H2BGFP marked SG cells can contribute to epidermal regeneration following injury.

However, since these experiments were performed “off Doxy” we were not able to rule out whether the SG H2BGFP LRCs themselves proliferated and contributed to this newly formed epidermis or whether other non-LRC SG cells “turned on” H2BGFP expression in the absence of Doxy. To address this, we repeated this experiment using 4 weeks chased H2BGFP labeled SGs. In this case, the host mouse with transplanted SG dermis was kept on Doxy treatment for the entire experiment; thus, only SG LRCs and their direct descendents would be marked by H2BGFP. At 30 and 40 days after transplantation, the H2BGFP label appeared to have been diluted out of some acinar SG structures (confirmed by K8 lumenal layer staining) but not all (Fig. 7M, 7N, 7Q, arrows). Moreover, some of these transplanted SGs appeared to be fused with the newly regenerated epidermis (Fig. 7M and 7Q). In some instances, SGs closer to the SG-epidermal connections were the ones lacking H2BGFP marked SG LRCs, suggesting that the LRCs of these SGs had been actively dividing and diluted out the nuclear H2BGFP label (Fig. 7N and 7Q, arrows). This observation was confirmed using Ki67 staining where we observed Ki67 positive cells present in SGs lacking LRCs as marked by green K5 membrane staining (Fig. 7O). Finally, we show that these SG LRCs themselves can also contribute to the regeneration of the epidermis as marked with K5 co-localization (Fig. 7P, arrows and inset). Staining for Ki67 illustrates further that SG LRCs near the basal layer of the newly regenerated epidermis, marked by basement membrane marker β4 integrin (CD104), can proliferate (Fig. 7R). In addition, SG LRCs can differentiate into cells of the suprabasal and granular layers of the epidermis as marked by K1 and loricrin co-localization, respectively (Fig. 7S and 7T). In total, we performed five chamber graft experiments with whole SGs where three were successful, consistently demonstrating the incorporation of H2BGFP marked SG cells into the epidermis. In conclusion, we have shown that under more favorable conditions, SG LRCs can divide and contribute to the different layers of the epidermis.

### Dissociated Sweat Gland Cells can Regenerate both Sweat Gland and Hair Follicle Appendages

**Figure 8:**
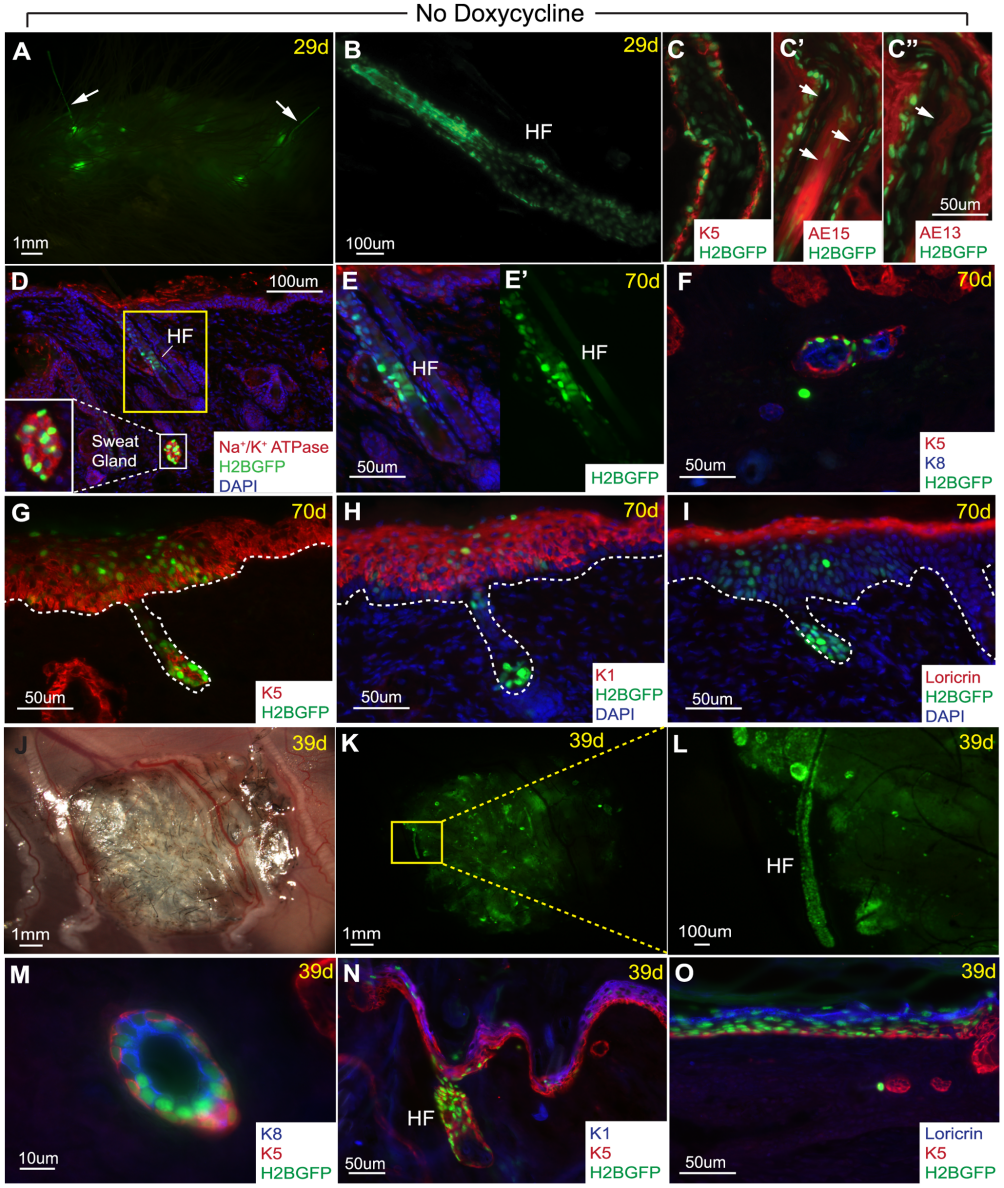
Dissociated sweat gland cells can regenerate sweat glands, hair follicles, and the epidermis. (A) Chamber graft of K5TetOff/TreH2BGFP dissociated 4 weeks chased sweat gland cells mixed with unmarked newborn dermal fibroblasts yield GFP positive hair-like fibers at 29 days. (B) Section through graft confirms the presence of H2BGFP positive hair follicles. (C) K5 staining marks the outer root sheath of this H2BGFP+ hair follicle. (C’) AE15 stains the inner root sheath and medulla, arrows. (C”) AE13 stains the hair shaft. (D) 70 days after transplantation, H2BGFP positive sweat gland structures were found, as confirmed by Na^+^/K^+^ ATPase expression (inset), in addition to H2BGFP positive hair follicle. (E) Magnification of the hair follicle in “D” with (E’) GFP single channel. (F) The sweat gland structures found also expressed K5 basal and K8 lumenal layer markers. (G) H2BGFP positive cells were also found in the K5 basal layer, (H) K1 suprabasal layer, and (I) loricrin marked granular layer of the newly regenerated epidermis. (J,K) In an independent experiment, 39 days after subcutaneous injections of 4 weeks chased unsorted dissociated cells from H2BGFP labeled SGs with unmarked newborn dermal fibroblasts, a cluster of GFP positive cells containing hair follicles were observed. (L) Magnification of a GFP+ hair follicle from panel “K”. (M) Sections show the presence of H2BGFP labeled SG structures expressing K8 lumenal layer marker. (N) H2BGFP positive cells are again also found in the basal, suprabasal, and (O) granular layers of the epidermis. White dotted lines mark dermal-epidermal interfaces.

Finally, since we were unable to passage and expand these K5TetOff/TreH2BGFP cells in culture, we could not probe their *in vitro* potential and subsequently use them for *in vivo* reconstitution assays. Instead, we used unsorted dissociated SG cells isolated directly from whole SGs. To further probe the regenerative potential of all SG cells, we dissociated 4 weeks chased, H2BGFP labeled, SGs into a single cell suspension after separation from the sole’s epidermis (as in Fig. 4B and 4C). Next, we performed chamber graft transplantation by mixing these unsorted H2BGFP marked dissociated SG cells with unmarked freshly isolated back skin dermal fibroblasts. Surprisingly, 29 days after transplantation, we observed several GFP positive areas under the epidermis with some of them connected to GFP positive hair-like fibers sticking out of the graft region (Fig. 8A). Indeed, analysis of sections from the graft area confirmed the presence of GFP positive hfs, likely originating from the transplanted unsorted H2BGFP labeled SG single cells suspension (Fig. 8B). These newly formed hfs were further characterized by immunofluorescence staining with several hair specific markers including K5 positive expression in the outer root sheath (Fig. 8C), AE15 expression in the inner root sheath and medulla (Fig. 8C’), and AE13 expression in the cortex of the hair shaft (Fig. 8C”). Interestingly, when we analyzed the graft 70 days after transplantation, we also found coexisting (on the same section) H2BGFP positive SG structures expressing SG markers: Na^+^/K^+^ ATPase and lumenal layer marker K8 (Fig. 8D, inset and 8F, respectively) in addition to GFP marked hfs (Fig. 8D–E’). This demonstrated that dissociated SG cells can still be flexible in their fate decision choices between SGs or hfs. Both these structures were found to coexist in the same region which is physiologically not observed in mice. Consistent with the SG graft data presented here, the single SG cells suspension can also contribute to the regeneration of differentiated epidermal layers marked by K5, K1, and loricrin (Fig. 8G–I).

In an independent experiment, we also subcutaneously injected unsorted dissociated cells from H2BGFP labeled SGs (chased for 4 weeks), after separation from the sole’s epidermis, in combination with freshly isolated dermal fibroblasts under the back skin of an immunocompromised “nude” mouse. This transplant was harvested 39 days after subcutaneous injection where we observed a cluster of hfs and GFP positive structures at the injection sites (Fig. 8J–K). Similar to the chamber graft experiment with dermal fibroblasts, we observed the presence of GFP positive hfs differentiated from the injected H2BGFP positive SG cells (Fig. 8L). In addition, we also found H2BGFP labeled SG structures containing both basal and lumenal layers marked by K5 and K8, respectively (Fig. 8M).These injected cells have again differentiated into the epidermis marked by K5, K1, and loricrin (Fig. 8N–O). Contamination from hfSCs are unlikely since hfs are absent in the palms where SGs are carefully dissected out. As a control, unsorted epidermal cells simultaneously isolated from the sole’s epidermis were also subcutaneously injected with freshly isolated dermal fibroblasts; however, no signs of H2BGFP marked hf formation were observed (data not shown) suggesting that these newly formed hfs and SGs are derived from SG cells.

## Discussion

Here, we demonstrate that cells with slow cycling characteristic exist in SGs as a scattered population localized in the SG basal layer of the proximal acinar region. As hair follicle LRCs have been previously described as SCs [24], we asked if these newly identified SG LRCs also possess bona fide stem cell characteristics *in vivo*. Although LRCs have been reported in both mouse and human SGs [19], their precise characterization and function has not been addressed so far. The K5TetOff/TreH2BGFP approach allows us to mark and isolate live SG LRCs *in vivo* for further characterization. Thus, we were able to localize LRCs in the basal layer of the proximal acinar part of SGs and demonstrate their myoepithelial characteristic by SMA co-expression. In addition, we demonstrated that SG LRCs specifically co-localize with p63 expression which has been shown to be specifically expressed in mammary gland myoepithelial cells [29]. Previous studies illustrated that p63 is not only essential for epithelial development, but is also important for epidermal self-renewal and differentiation [30], [40]. In addition, p63 is believed to be a marker of corneal and epidermal SCs [41].

Recently, some similar findings regarding SG LRCs were reported by Lu et al. [18], however, they did not further characterize this SG LRC population or address their *in vivo* function. Instead, they employed elegant systems, previously published in mammary glands [42], to identify and characterize distinct SC populations in the basal and lumenal layers of SGs [18]. In our study, we used a different approach and focused predominantly on basal, myoepithelial LRCs (GFP+/α6+) after 4 weeks of chase. Although we were able to characterize this population of SG LRCs, this genetic approach did not allow us to study the lumenal layer of SGs in more detail.

### The Proximal Acinar SG Region only Contributes to its Own Structure during Homeostasis and Wound Healing

As an alternative and parallel approach, we used genetic K15CrePR *in vivo* systems to mark cells specifically in the proximal acinar part of SGs including SG LRCs in the basal layer (Fig. 3C). However, these K15 labeled cells did not specifically mark SG LRCs, but had also marked cells of the lumenal layer in the proximal acinar part of SGs as illustrated with K15-GFP reporter mice (Fig. 3D and 3E). This data was confirmed by FACS analysis of SGs from adult K15-GFP reporter mice where we observed an approximate 1∶1 proportion of GFP marked basal and lumenal cells (Fig. 3F). Although our finding differs from data recently published by Lu et al., this discrepancy could be attributed to the use of different time points for labeling these K15 progenitors. In our case, we marked them by RU treatment for K15CrePR systems or analyzed them in K15-GFP reporter mice during adulthood at/or after P21 (after SG morphogenesis was completed), whereas Lu et al. labeled them at an early postnatal time point between P5 to P9 (during SGs morphogenesis) and analyzed them at P22 [18]. Thus, it appears that this K15 promoter has different specificity during and after SG morphogenesis.

Since the K15CrePR system permanently marks K15 expressing cells and its progeny, we used it to evaluate the contribution of K15 marked acinar cells in overall SG and skin homeostasis. Our results demonstrate that K15 labeled cells localized exclusively in the acinar part of SGs and contributed long term to only the proximal glandular part, but not to the homeostasis of the distal SG ducts or the surrounding epidermis (Fig. 3G). Furthermore, we also show that typical wound stimulation surprisingly did not activate these proximal acinar SG cells to participate in epidermal healing (Fig. 6A–C). Instead, we observed that only SG duct cells proliferated (Fig. 6E). In general, these results support previously published wound healing data in human SGs by Lobitz et al. [15] as well as mouse SGs by Lu et al. [18] which showed the contribution of SG duct cells in wound healing. However, since Lu et al. used a different system, Sox9CreER/RosaLacZ or YFP, they could not rule out fully if these contributing cells are in fact coming only from the duct or from the upper acinar part of SGs as well. In addition, they also used the K15CrePR/R26LacZ approach; however, they specifically labeled only lumenal SG cells in the glandular part after early postnatal RU treatment and could not address whether basal layer myoepithelial cells could respond to the injury. Although our results are consistent, using later adult postnatal RU applications in K15CrePR/R26LacZ mice allowed us to mark both basal and lumenal layers of the proximal acinar part of SGs. Therefore, we were able to extend this conclusion to both SG layers and make the statement that not only lumenal but basal cells as well from the SG proximal acinar region do not contribute to wound healing of the epidermis. Collectively, previous reports together with our findings emphasize that both layers of SG cells remain quiescent in the proximal acinar region during wound healing and only SG ductal cells were able to proliferate. In the future, it still remains to be addressed which duct layer, lumenal or basal, can contribute to wound healing and cells from which layers are able to fully trans-differentiate into epidermal cells long term.

### SG LRCs Possess Multipotency and Stem Cells Characteristic *in vivo* and has Potential to Trans-Differentiate into the Epidermis under Prolonged Isolated Wound Healing

As we demonstrated here, K15CrePR/R26LacZ labeled cells in the acinar part of SGs appear to be generally slow cycling, but these cells were able to selectively maintain and participate in the long term homeostasis of the glandular part of SGs (Fig. 3G). Thus, it suggests that at least part of this SG structure contains cells with SCs characteristic that can maintain this glandular portion. Although K15 labeled cells overlap with SG LRCs in the basal layer of SGs, it also marked cells of the lumenal layer (Fig. 3C–E) preventing us from determining whether SG LRCs themselves possess stem cells characteristic. In addition, we show that SG LRCs survive long term and persists for more than 20 weeks of chase illustrating their extreme slow cycling properties. Although the H2BGFP label persists for such a long period of chase, we demonstrate that its intensity slowly diminishes as these SG LRCs slowly divide over time (Fig. S1).

To assess the stem cell properties of SG LRCs *in vivo*, we had to challenge our system further since regular wound healing conditions failed to provoke SG acinar cells to contribute to epidermal regeneration. Under this special wound condition where we gave SG cells an advantage by preventing wound closure from the surrounding epidermis, we observed that 4 weeks chased H2BGFP labeled SG LRCs can proliferate and trans-differentiate into all epidermal layers (Fig. 7P, 7S, 7T). In this experiment, animals were kept on continuous Doxy treatment allowing us to distinguish SG LRCs from other cells and confirm that SG LRCs themselves are multipotent.

Together, our results remain in agreement with previously published results on human, mouse and porcine [13], [14], [16], [17], [18], [43], demonstrating that in general, SG cells can respond and re-epithelialize the skin after wounding. However, for the first time, we show that under more favorable, isolated, and prolonged wound healing conditions, normally quiescent myoepithelial SG LRCs can contribute to and reconstitute a stratified epidermis. Thus, we demonstrate that under favorable conditions, these relatively quiescent SG LRCs can be activated and work as an alternative source of cells confirming that these cells are multipotent with SC characteristics *in vivo*.

### Purification and Characterization of Basal Layer Myoepithelial SG LRCs from the Acinar Sweat Gland Region

To further characterize these SGSCs, we used the K5TetOff/TreH2BGFP approach to localize and isolate SG myoepithelial LRCs from the proximal acinar part of SGs. We purified SG LRCs and adjacent basal layer cells representing SG non-LRCs, which were predominantly composed of basal layer cells from the acinar and ductal regions. Although all SG LRCs showed myoepithelial characteristics co-expressing SMA and p63, only a fraction of SMA positive cells were LRCs while the remaining majority of SMA positive cells did not display label retaining characteristics (Fig. 2F). Thus, in our experimental setup, we specifically targeted and purified SG LRCs which represented approximately 30 to 40% of all basal SG cells attached to the basement membrane. Consequently, our SG LRCs purification strategy was different from the strategy recently published by Lu et al., where they purified all myoepithelial cells including those without LRC characteristic as well as lumenal cells in both glandular and ductal parts to identify and characterize distinct unipotent stem cell populations in SGs. They used Abs against α6(CD49) and β1 (CD29) integrins, markers previously published for mammary gland SCs purification [44], [45], in conjunction with K14-H2BGFP mice which marked basal cells with high GFP intensity and lumenal or suprabasal layer cells with lower GFP intensity. In contrast, we focused more selectively on basal myoepithelial cells with LRCs and non-LRCs characteristic. Our microarray data revealed gene expression changes in both basal populations, SG LRCs and SG non-LRCs, when compared to the sole’s epidermis. This allowed us to identify the expression of various ion channels important for SG function including the Na^+^/K^+^ ATPase pumps, Atp1a1, Atp1b1, and Atp1b3. In addition, vimentin, which was previously published as a marker of the human SG myoepithelium [34], was found up-regulated in SG LRCs and SG non-LRCs populations. Interestingly, we found components of BMP signaling including Bmpr1a, Bmpr2, Smad5, Id2, Id3 and Decorin to be up-regulated in the SG when compared to the sole’s epidermis (Fig. 5B) suggesting a quiescent behavior of those populations *in vivo*. This is consistent with a well-known role of BMP signaling in maintaining quiescence in several adult stem cell populations including hair follicle, hematopoietic, intestinal and neural stem cells [46], [47], [48], [49], [50], [51]. Although the direct role of BMP signaling in adult SGSCs has not been investigated yet, we were able to confirm the functional requirement of Bmpr1a during SG formation (Fig. 5J and 5L). In the future, it will be important to develop new genetic tools to specifically address further questions about the function of several newly identified genes in SGs to better understand their role in SG biology both during development and in adult SG homeostasis.

### Plasticity of Sweat Gland Cells to Reconstitute Sweat Glands and Hair Follicles in vivo

In our reconstitution assay, we showed that dissociated SG cells were able to generate H2BGFP positive SG structures expressing SG markers K8 and Na^+^/K^+^ ATPase (Fig. 8D, 8F, 8M). Surprisingly, we also observed the formation of a few hair follicles with fully differentiated hair shaft fibers (Fig. 8A–E’). In addition, both SG and hf appendages were found to coexist in the same region which is physiologically not observed in the mouse’s sole (only SGs) nor back skin (only hfs). Since this finding was unexpected, we tested for possible contamination from surrounding paw’s epidermis which could potentially be a source of keratinocytes capable of generating hair under the inductive properties of freshly isolated GFP negative newborn dermal fibroblasts. For our control, we used unsorted cells isolated from the attached 4 weeks chased sole’s epidermis mixed with freshly isolated unmarked dermal fibroblasts and did not observe any H2BGFP positive hf formation. Thus, it is unlikely that the H2BGFP marked hfs resulted from contaminating keratinocytes, however, fewer cells were used in this control experiment when compared to the SG reconstitution assay. Instead, an alternative explanation would be that some SG cells were able to adopt this new hf fate. This observation might suggest that some SG cells still possess plasticity to choose between their final fate decisions dependent on surrounding environmental cues. This raises additional questions to be addressed in the future about signals which promote SGs but not hair fate. Surprisingly in this assay, even when we used freshly isolated dermal fibroblasts from back skin, which normally induce hf formation, the inductive signals from these fibroblasts still induced SG formation in addition to hfs. Thus, it is possible that inductive signals are similar during the development of these appendages. In fact, Plikus et al. published that overexpression of a BMP signaling inhibitor, Noggin, resulted in trans-differentiation of SGs into hfs [52]. Since we have shown here as well that BMP signaling is critical for SG formation, it will be interesting to address how fine tuning of this pathway might modulate its fate decision in the future.

Taken together, we have explored the role of LRCs in SGs and were able to localize them to the basal layer myoepithelial cells of the proximal acinar region. We were able to isolate these SG LRCs which allowed us to further characterize them and determine their gene expression profile. Among these genes, a number of BMP signaling genes were identified and we demonstrated the requirement of this signaling pathway in SG formation. We propose that SG LRCs are the SC population required for the maintenance and homeostasis of the SG skin appendage. Our data suggests that at least one distinct stem cell population exists in the proximal acinar region of SGs, which contains relatively quiescent cells contributing only to their own glandular structures during homeostasis and typical wound healing. In fact, our results are in agreement with previously published observations in human SGs, where SG ducts were completely or partially injured in the dermis [16]. Interestingly, they observed that the deep portion of SGs maintained their quiescence and survived similar to the acinar part containing SGSCs in our study. In contrast, the ductal part of human SGs were not able to rebuild the lower acinar part of SGs *in vivo*, but had instead slowly disappeared [16]. Moreover, we demonstrated that SG LRCs in the acinar compartment in fact possess multipotency and SCs characteristic *in vivo* having the potential to trans-differentiate into the epidermis under prolonged isolated wound healing conditions. Finally, our data also suggest plasticity of SG cells to reconstitute both SGs and hfs *in vivo*.

## Materials and Methods

### Immunohistochemistry and Immunofluorescence Staining

All frozen sections were fixed in 4% Paraformaldehyde. Tissue sections were stained with hematoxylin and eosin for H&E visualization. For LacZ visualization, frozen sections were fixed in 0.2% Glutaraldehyde for 1 min, washed with PBS, and stained with 1 mg/ml X-gal overnight at 37°C. For immunofluorescence staining, sections were permeabilized with 0.1% Triton X-100 for 10 min and blocked in 0.1% Triton-PBS, 0.5% goat serum, and 0.1% BSA for 1 h at room temperature. Primary antibodies were incubated in the blocking buffer overnight at 4°C and washed with PBS. Secondary antibodies were incubated in 0.1% BSA for 1 h at room temperature. The following primary antibodies were used: K14 (1∶200; gift from E. Fuchs Lab), CD104 (1∶100; BD Pharmingen, 553745), K8 (TROMA-1, 1∶100; Developmental Studies Hybridoma Bank), K18 (1∶200; gift from E. Fuchs Lab), p63α (H-129, 1∶100; Santa Cruz Biotech, sc-8344), SMA (1∶200; Sigma, A5228), Laminin (1∶100; Thermo Scientific, RB-082-A1), K15 (1∶100; Thermo Scientific MS-1068-P1), Na^+^/K^+^ ATPase (1∶300; abcam ab58475), K5 (1∶300; gift from C. Jamora), Ki67 (1∶200; Leica, NCL-Ki67p), P-Smad1/5/8 (1∶50; Cell Signaling, 9511), BrdU (1∶200; abcam ab6326), K1 (1∶300; gift from C. Jamora), Loricrin (1∶300; gift from C. Jamora), AE15 (1∶100; Santa Cruz Biotech, sc57012), AE13 (1∶100; Santa Cruz Biotech, sc80607). Secondary antibodies: Rabbit anti-Rat TRITC (1∶300; Sigma T4280), Goat anti-Rabbit TRITC (1∶300; Sigma T6778), Goat anti-Mouse TRITC (1∶300; Sigma T6528), Alexa 594 Goat anti-Chicken (1∶500; Invitrogen A11042), Alexa 488 Goat anti-Chicken (1∶500; Invitrogen A11039), Goat anti-Rabbit FITC (1∶300; Sigma F9887), Alexa 350 Goat anti-Rat (1∶150; Invitrogen A21093), Alexa 350 Donkey anti-Rabbit (1∶150; Invitrogen A10039).

### Isolation of SG LRCs and Sole’s Epidermal Basal Cells

K5TetOff/TreH2BGFP animals were fed 1 mg/g doxycycline food for 4 weeks starting around P21–28. H2BGFP+ sweat glands were dissected out with its surrounding sole’s epidermis from the fingertips of 20–30 mice and treated with 1000 U/ml Collagenase type I for 1 h at 37°C with shaking. Sweat glands were mechanically separated from its epidermis and treated further with 1000 U/ml Collagenase type I and 500 ug/ul Hyaluronidase (Sigma) for 1 h at 37°C with shaking. Purified sole’s epidermis and SGs were independently washed with DPBS and digested in 0.25% Trypsin-EDTA for 20 min at 37°C with shaking. Neutralize and filter cells through a 40 um cell strainer.

### FACS

For FACS, isolated 4 weeks chased H2BGFP labeled sweat gland cells were stained with a primary antibody: PE conjugated anti-α6 integrin (CD49f) (1∶200; BD Pharmingen) for 30 min and sorted using the FACS Aria II cell sorter (BD, Bioscience) for H2BGFP+/α6+ and H2BGFP−/α6+ populations. Cells were collected in RNAprotect Cell Reagent (Qiagen) for later RNA isolation. Similarly, FACS analysis were performed on isolated K15-GFP labeled sweat gland cells, stained with the primary antibody against anti-α6 integrin as described above.

### Chamber Graft

A silicon chamber was implanted onto the backs of immunocompromised “nude” mice with a full-thickness skin wound as previously described [39]. 4 weeks chased whole H2BGFP sweat glands were dissected out with its surrounding sole’s epidermis from the fingertips and treated with 1000 U/ml Collagenase type I for 1 h at 37°C with shaking. After separation from the sole’s epidermis, purified dermis with remaining sweat glands were transplanted into the humidified silicone chamber. The upper chamber was removed 2 weeks after transplantation and the bottom half is removed 3 weeks after transplantation as the skin is healing. The nude mice were either fed regular mouse diet or doxycycline food after transplantation for the duration of the experiments.

Similarly, dissociated unsorted H2BGFP marked 4 weeks chased SG single cell suspension after separation from the sole’s epidermis was mixed with freshly isolated unmarked newborn dermal fibroblasts (approximately 6 million cells total) at a 1∶1 proportion and injected into the chamber. Mice were sacrificed and samples from the graft regions were taken for GFP+ expression and tissue analysis. All mice work was conducted according to the Institutional Animal Care and Use Committee (IACUC) at the University of Southern California. The protocols (No. 11306 and 11325) were approved by the IACUC Committee. All surgery was performed under either isoflurane or ketamine anesthesia and all efforts were made to minimize suffering with analgesics (Buprenex prior and post surgery was administrated).

### Subcutaneous Injections

Dissociated unsorted SG single cell suspension labeled with H2BGFP after 4 weeks of chase after separation from the sole’s epidermis was mixed with freshly isolated unmarked newborn dermal fibroblasts at a 1∶1 proportion and injected subcutaneously underneath the back skin of an immunocompromised “nude” mouse (approximately 1.5 million cells per spot).

### Confocal Microscopy of Sweat Gland LRCs and 3D Reconstruction of Sweat Glands

Tissue was imaged on a Zeiss (Carl Zeiss, LLC) Axiovert 200 inverted microscope with an LSM 510 meta confocal scan head using a 40×/1.2NA water immersion lens. Tissue was dissected, stained and placed in a 35 mm glass bottom tissue culture dish (MatTek Corporation, Asland, MD). Tissues were submerged in deionized water to minimize refractivity. Histone 2B GFP and TRITC stained laminin (1∶100; Thermo Scientific, RB-082-A1) were visualized using conventional confocal imaging using argon laser lines at 488 nm and 543 nm, respectively. DAPI was imaged with 2-photon excitation using a Coherent Chameleon (Coherent Inc, Santa Clara, CA), pulsed laser tuned to 800 nm. Images were collected at 0.22 µm in plane (xy) and optically sectioned at 2 µm (in z). 3D reconstruction and visualizations were performed in ImageJ (http://rsbweb.nih.gov/ij/), Fiji (http://fiji.sc/wiki/index.php/Fiji), Avizó 6.3 (VSG, Burlinton, MA) and Vaa3D (http://www.vaa3d.org/).

### RNA Isolation and qPCR

Total RNAs were purified from FACS-sorted SG LRCs, SG non-LRCs, and the basal layer of the sole’s epidermis using Qiagen’s RNeasy Micro kit according to the manufacturer’s instructions. Equal amounts of RNA were reverse transcribed using the Superscript III First-Strand Synthesis System (Invitrogen) according to the manufacturer’s instructions. cDNAs were amplified by PCR and used in triplicate for each qPCR sample primer set with all primer sets designed to work under the same conditions. Real-time PCR amplification of particular genes of interest was performed using an Applied Biosystems 7900HT Fast Real-Time PCR System and the fold difference between samples and controls were calculated based on the 2^−ΔΔCT^ method, normalized to β-actin levels.

### Microarray Analysis

Total RNAs from FACS of SG LRCs (GFP+/α6 integrin+), SG non-LRCs (GFP−/α6 integrin+), and sole’s epidermis (α6 integrin+) were purified using a RNeasy Micro Kit (Qiagen, Valencia California, United States), and quantified (Nanodrop, United States) for two separate microarray analysis from two independent biological samples. RNA 6000 Pico Assay (Agilent Technologies, Palo Alto, California, United States) was used for RNA quality check. Amplification/labeling were performed on 50 nanogram (ng) and 250 ng to obtain biotinylated cRNA (Ovation™ RNA Amplification System; Nugen, San Carlos, California, United States and Ambion Kit; Affymetrix, Santa Clara, California, United States, respectively), and either 3.75 μg or 5.5 μg ssDNA were used for fragmentation, labeling and hybridization. Hybridization was performed at 45°C for 18 h to Mouse Gene 1.0 ST array (Affymetrix, Santa Clara, California, United States). Processed chips were read by GeneChip Scanner 3000 7G (Genomics Core Facility, Children’s Hospital Los Angeles, Los Angeles, California, United States). The raw expression intensity data was imported into Partek Genomic Suite v6 (Partek Inc., St. Louis, MO, United States). The data was pre-processed using the RMA algorithm with the default Partek setting. Following fold change calculations, differentially expressed gene (DEG) lists containing probe sets with 2-fold intensity changes in either direction were generated. Common DEG list was generated by comparing the DEG list of the sweat gland LRCs experiment and GFP-α6+ sweat glands experiment to the sole’s epidermis. Functional annotation of the DEG list was carried out using the “Database for Annotation, Visualization and Integrated Discovery” (DAVID). The microarray data are available in a public GEO database with an accession number (#GSE49011).

## Supporting Information

Figure S1 **Sweat gland LRCs possess slow cell cycle dynamics but are non post-mitotic cells.** Hair follicle bulge at (A) 4 weeks, (B) 10 weeks, (C) 15 weeks and (D) 20 weeks of chase with doxycycline. 10× magnification of sweat glands at (E) 4 weeks, (F) 10 weeks, (G) 15 weeks and (H) 20 weeks of chase with doxycycline. 20× magnification of sweat glands at (I) 4 weeks, (J) 10 weeks, (K) 15 weeks and (L) 20 weeks of chase with doxycycline. Exposure time of all 10× images is 800 ms and 20× images is 160 ms. White dotted lines mark dermal-epidermal interfaces.
(TIF)

Figure S2 **Validation of genes identified in the microarray analysis by real time PCR.** Using SG LRCs as the baseline, we confirmed an up-regulation of Bgn, Mmp2, and Timp2 in SG non-LRCs (α6+ basal layer cells) when compared to GFP+/α6+ SG LRCs in either 2 or 3 independent biological samples. Representative data from one is shown. Error bars represent standard deviation.
(TIF)

Table S1 **Common DEG list for both SG LRCs and SG non-LRCs.** Functionally categorized list of genes commonly identified in both SG LRCs (GFP+/α6+) and SG non-LRCs (GFP−/α6+) when compared to the basal layer of the sole’s epidermis.
(XLSX)

Table S2 **Unique DEG list for SG LRCs.** Functionally categorized list of genes identified in SG LRCs (GFP+/α6+) when compared to the basal layer of the sole’s epidermis.
(XLSX)

Table S3 **Unique DEG list for SG non-LRCs.** Functionally categorized list of genes identified in the basal layer SG non-LRCs (GFP−/α6+) when compared to the basal layer of the sole’s epidermis.
(XLSX)

Movie S1 **Reconstruction of 3-dimensional (3D) structure of 4 weeks chased sweat glands.** 3D reconstruction of whole 4 weeks chased sweat glands stained with basement membrane marker, laminin (red), and counterstained with DAPI (blue).
(MOV)
