# Supplementary figures and images for: Label Retaining Cells (LRCs) with Myoepithelial Characteristic from the Proximal Acinar Region Define Stem Cells in the Sweat Gland
Source: PLoS One. 2013 Sep 18;8(9):e74174. doi: 10.1371/journal.pone.0074174 (PMC3776797; doi:10.1371/journal.pone.0074174)

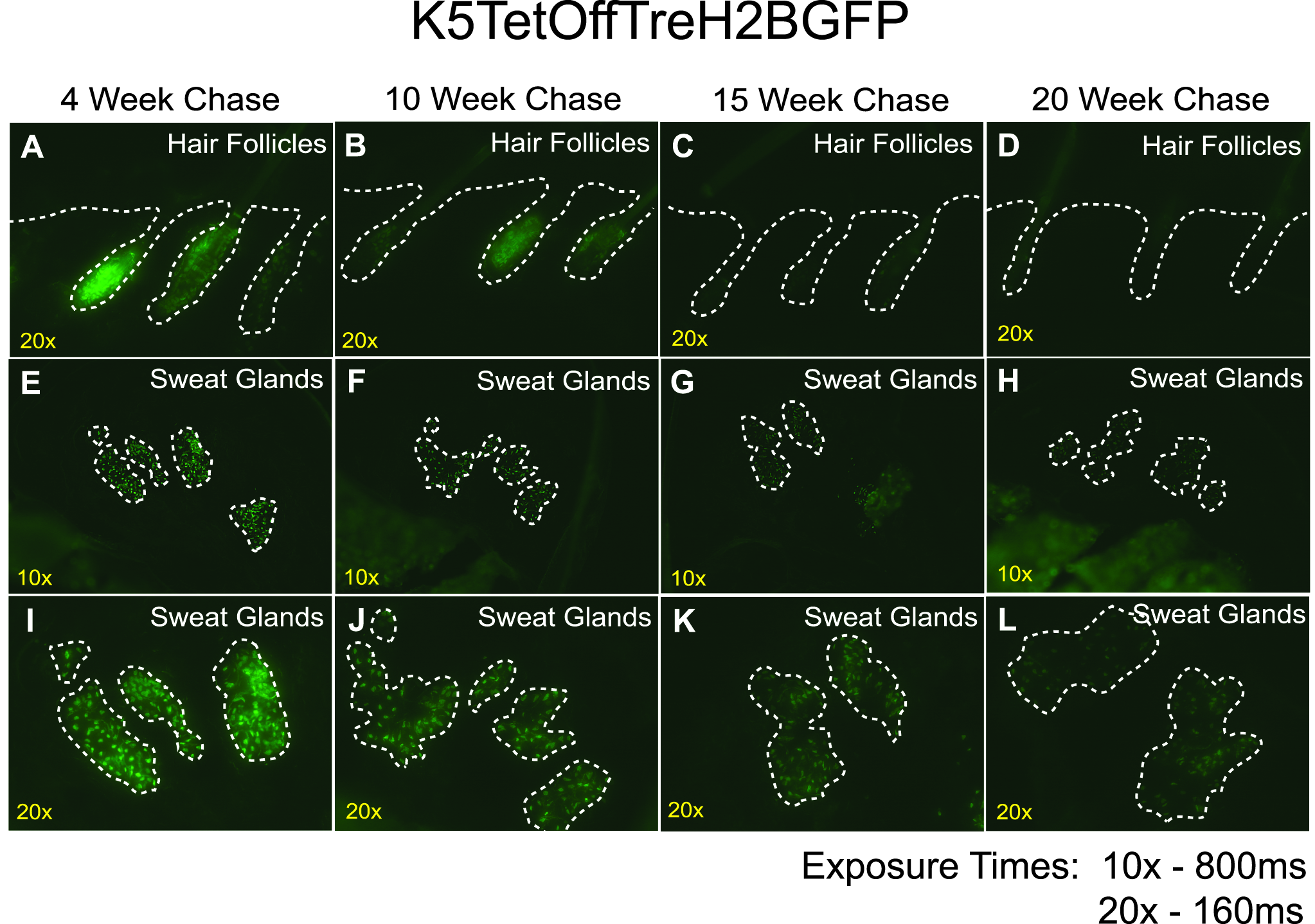

Supplement: Figure S1 — Sweat gland LRCs possess slow cell cycle dynamics but are non post-mitotic cells. Hair follicle bulge at (A) 4 weeks, (B) 10 weeks, (C) 15 weeks and (D) 20 weeks of chase with doxycycline. 10× magnification of sweat glands at (E) 4 weeks, (F) 10 weeks, (G) 15 weeks and (H) 20 weeks of chase with doxycycline. 20× magnification of sweat glands at (I) 4 weeks, (J) 10 weeks, (K) 15 weeks and (L) 20 weeks of chase with doxycycline. Exposure time of all 10× images is 800 ms and 20× images is 160 ms. White dotted lines mark dermal-epidermal interfaces. (TIF) [file pone.0074174.s001.tif]

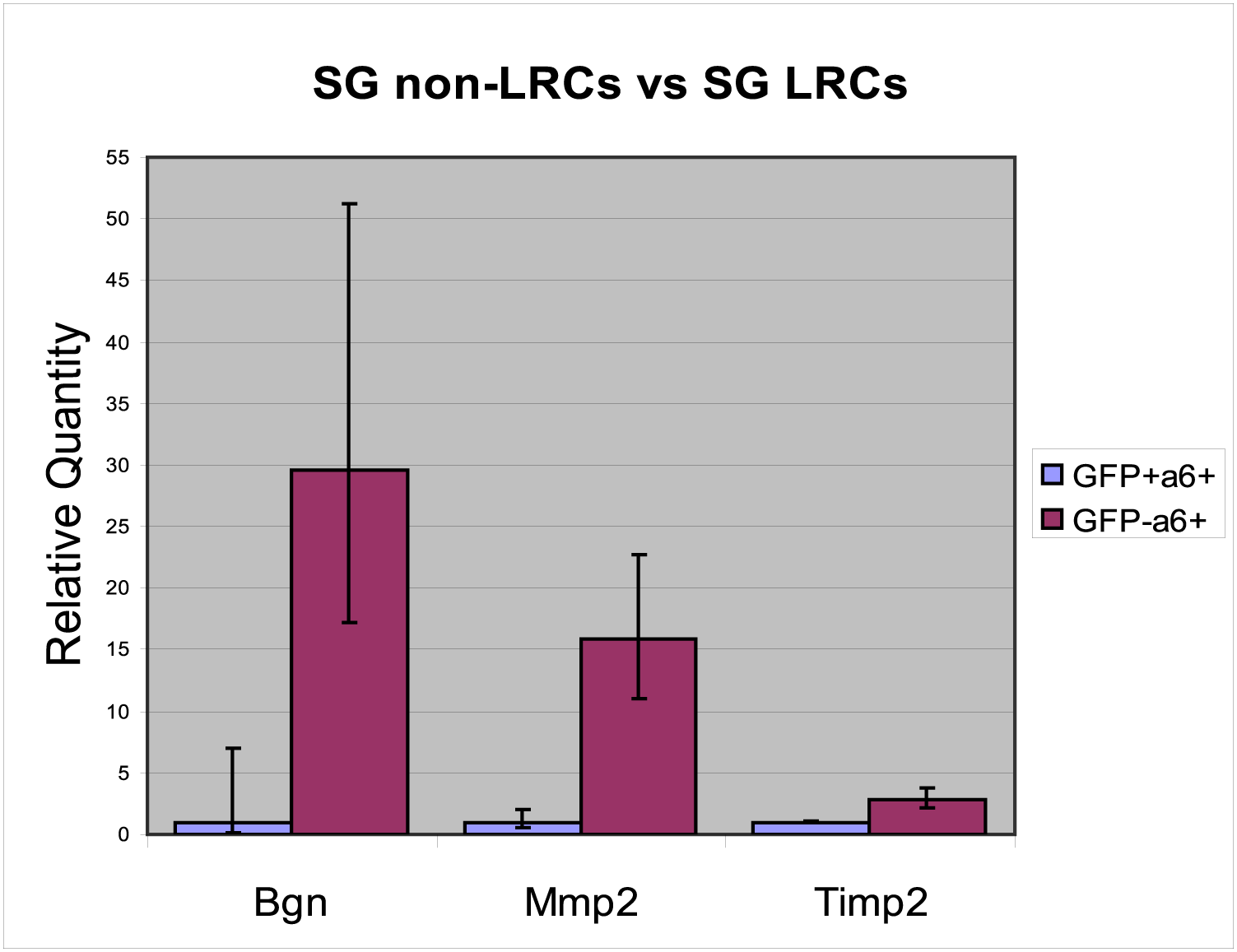

Supplement: Figure S2 — Validation of genes identified in the microarray analysis by real time PCR. Using SG LRCs as the baseline, we confirmed an up-regulation of Bgn, Mmp2, and Timp2 in SG non-LRCs (α6+ basal layer cells) when compared to GFP+/α6+ SG LRCs in either 2 or 3 independent biological samples. Representative data from one is shown. Error bars represent standard deviation. (TIF) [file pone.0074174.s002.tif]
